# Triterpenoid-Rich Extract from *Antrodia camphorata* Improves Physical Fatigue and Exercise Performance in Mice

**DOI:** 10.1155/2012/364741

**Published:** 2012-07-05

**Authors:** Chi-Chang Huang, Mei-Chich Hsu, Wen-Ching Huang, Huei-Ru Yang, Chia-Chung Hou

**Affiliations:** ^1^Graduate Institute of Sports Science, National Taiwan Sport University, Taoyuan 33301, Taiwan; ^2^Department of Sports Medicine, Kaohsiung Medical University, Kaohsiung 80708, Taiwan; ^3^Dong Jyu Biotechnology Corporation, Taipei 10683, Taiwan, Taiwan; ^4^Graduate Institute of Athletics and Coaching Science, National Taiwan Sport University, Taoyuan 33301, Taiwan

## Abstract

*Antrodia camphorata* (AC) is an endemic mushroom that grows in Taiwan. We investigated the fatigue-alleviating effects of AC on endurance capacity in swim-exercised and weight-loading mice. Male Institute of Cancer Research (ICR) strain mice from 3 groups (*n* = 10 per group in each test) were orally administered AC fruiting body extract for 7 days at 0, 50, and 200 mg/kg/day, designated vehicle, AC-50, and AC-200, respectively. Trend analysis revealed that AC treatments increased grip strength. AC dose-dependently increased swim time, blood glucose, and muscular and hepatic glycogen levels and dose-dependently decreased plasma lactate and ammonia levels and creatine kinase activity. The increase in swimming endurance with AC administration was caused by an increase in liver and muscle glycogen deposition. *A. camphorata* may have potential for use in ergogenic and antifatigue activities.

## 1. Introduction

Fatigue is characterized as physical and/or mental weariness resulting in negative impacts on work performance and exercise intensity, family life, and social relationships [1]. Fatigue can be classified as secondary, physiologic, or chronic. Secondary fatigue results from disturbed sleep, depression, excess exertion, and medication side effects. Physiological fatigue is caused by inadequate rest, physical effort or mental strain [2]. Chronic fatigue syndrome involves a persistent unexplainable fatigue lasting for more than 6 months, but the etiology remains unclear [3]. Long-term physical and mental fatigue leads to health damage and chronic fatigue [4]. Physical fatigue is also called peripheral fatigue and may be accompanied by deterioration in performance [5]. Two mechanisms, oxidative stress and exhaustion, play an important role in physical fatigue [6]. Hard work or intense exercise can lead to the production and accumulation of excess reactive free radicals, which results in oxidation stress injury to the body. Exhaustion theory suggests that energy source depletion and excess metabolite accumulation lead to fatigue [7]. However, several studies have shown that exogenous antioxidants can reduce exercise-induced oxidative stress [8]. Research in specific nutrients or herbal supplements is needed to find agents that reduce metabolite production and/or improve energy utilization.

The fungus *Antrodia camphorata *is a traditional Chinese remedy used by the Taiwan aboriginal community for discomforts caused by alcohol drinking or exhaustion. It grows only on the unique, local, large, evergreen broad-leaved tree* Cinnamomum kanehirae *Hayata (Lauraceae). The fruiting bodies of *A. camphorata* are well-known but expensive medicinal material that are used in Asian countries to treat several diseases such as liver disease, tumors, intoxication, and abdominal pain [9]. The chemical and pharmacological properties of *A. camphorata* extracts from its mycelium, cultivation filtrate, and fruiting bodies have been investigated. These studies demonstrated that *A. camphorata* has extensive bioactivities including anticancer [10], anti-inflammation [11], immunomodulation [12], and hepatoprotection [13] activities.

Herbal medicines and natural compounds have been investigated as an important resource for postponing fatigue, accelerating the elimination of fatigue-related metabolites, and improving athletic ability. For a long time *A. camphorata* has been considered a potent remedy for regulating the body balance, with few adverse effects. Although more detailed studies are needed to confirm the traditional effects in light of rational bioactivity function, *A. camphorata* may be an antifatigue herbal supplement candidate. We examined the antifatigue activities of ethanol extracts of AC fruiting bodies in mice. We further examined the possible mechanism and the active components to determine a scientific basis for use of this natural species for fatigue. 

## 2. Materials and Methods

### 2.1. Plant Material

 Fresh *A. camphorata* fruiting bodies were cultivated on *Cinnamonum Kanehirai* Hayata and provided by Dong Jyu Biotechnology Corp. (Taipei). All materials were identified by the Food Industry Research and Development Institute (Hsinchu, Taiwan). A voucher specimen was deposited in the Graduate Institute of Sports Science, National Taiwan Sport University (Taoyuan, Taiwan).

### 2.2. Extract Preparation and Identification of Index Compounds

Fresh *A. camphorata* fruiting bodies were freeze-dried and ground into powder. The dried powder (45 g) was extracted 3 times with ethanol under reflux. After filtration, the solvent was concentrated by use of a rotary evaporator to obtain the ethanol extract (15 g). Total extract was partitioned with ethyl acetate to yield an ethyl acetate fraction (14.8 g), which was chromatographed on an MCI CHP 20P column eluted with H_2_O-MeOH gradient to yield 1–7 fractions. After recrystallization with acetone, antcin K (1) was isolated from fraction 2. Fraction 3 was subsequently separated on silica gel column eluted with CH_2_Cl_2_/MeOH gradient to give antcin C (2), antcin H (3), and dehydrosulphurenic acid (4). Fraction 4 was purified by ODS gel (H_2_O-MeOH gradient) to obtain antcin I (5). Chromatography of fraction 5 over a Sephadex LH-20 column with H_2_O-MeOH yielded antcin B (6) and 15*α*-acetyl-dehydro-sulphureic acid (7). All chemical structures were identified by nuclear magnetic resonance and mass spectral analyses and were in agreement with published data [14].

### 2.3. Animals and Experiment Design

Specific pathogen-free male ICR mice (5 weeks old) were purchased from BioLASCO (A Charles River Licensee Corp., Yi-Lan, Taiwan). All animals were given a standard laboratory diet (no. 5001; PMI Nutrition International, Brentwood, MO, USA) and distilled water *ad libitum *and housed at room temperature (23 ± 1°C) with a 12 h light/12 h dark cycle (lights on from 6:00 AM to 6:00 PM). All animal experiments adhered to the guidelines of the Institutional Animal Care and Use Committee (IACUC) of National Taiwan Sport University (NTSU). The IACUC ethics committee approved this study under the protocol IACUC-9903. 

Mice were divided into 3 groups (*n* = 10 per group in each test) for treatment: (1) vehicle, (2) 50 mg/kg ethanol extract of AC fruiting body (AC-50), and (3) 200 mg/kg ethanol extract of AC fruiting body (AC-200). Vehicle or AC extract was given once by oral gavage for 7 days each. The control group received the same dose of vehicle. 

### 2.4. Forelimb Grip Strength

A low-force testing system (Model-RX-5, Aikoh Engineering, Nagoya, Japan) was used to measure forelimb grip strength in mice. The force transducer equipped with a metal bar (2 mm in diameter and 7.5 cm in length) was used to measure the amount of tensile force from each mouse. As described in [15], we grasped the mouse at the base of the tail and lowered it vertically toward the bar. The mouse was pulled slightly backwards by the tail while the 2 paws (forelimbs) grasped the bar, which triggered a “counter pull.” This grip strength meter recorded the grasping force in grams. Before AC administration, all mice were trained to perform this procedure for 3 days. The 3 groups did not differ in performing the activity. Grip strength was measured 1 h after the last treatment administration. The maximal force (grams) exerted by the mouse counter pull was used as forelimb grip strength. 

### 2.5. Forced Swimming Test

The protocol was adapted from a previous study with some modifications [16]. Mice were pretreated with vehicle, AC-50, or AC-200 for 7 days and 1 h after the last treatment administration and underwent an exhaustive swimming test. The mice were placed individually in a columnar swimming pool (length 65 cm and radius 20 cm) with 40 cm water depth maintained at 37 ± 1°C. A weight equivalent to 5% of body weight was attached to the root of mouse tail, and endurance for each mouse was measured as swimming times recorded from the beginning of the time in the pool to exhaustion. The swimming period was considered the time spent floating, struggling, and making necessary movements until exhaustion and possible drowning. When the mice were unable to remain on the water surface, they were considered exhausted. 

### 2.6. Determination of Blood Biochemical Variables

We evaluated the effects of AC on plasma lactate, ammonia, and glucose levels and creatine kinase (CK) activity after exercise. A 15 min swimming test was performed 1 h after the last treatment administration. Blood samples were collected from the submandibular duct of pretreated mice after the 15 min swimming test. The plasma was prepared by centrifugation at 1500 ×g, 4°C for 10 min. Lactate, ammonia, and glucose levels and CK activity were determining by use of an autoanalyzer (Hitachi 7060, Hitachi, Japan).

### 2.7. Tissue Glycogen Determination

To investigate whether AC increases glycogen deposition in target tissues, mice were pretreated with vehicle, AC-50, and AC-200 for 7 days and 1 h after the last treatment administration and the liver and muscle were excised and weighed for tissue glycogen level analysis. The muscular and hepatic glycogen levels were measured as described in [17]. For each mouse, 100 mg of liver and muscle was finely cut, weighed, and homogenized in 0.5 ml cold perchloric acid. The homogenate was centrifuged for 15 min at 15000 ×g at 4°C. The supernatant was carefully decanted and kept on ice. A standard glycogen (Sigma) or tissue extract, 30 *μ*L, was added to 96-well microplates, and iodine-potassium iodide reagent, 200 *μ*L, was added to each well for binding iodine to glycogen. An amber-brown compound developed immediately after the reaction. Absorbance was measured at wavelength 460 nm with use of an ELISA reader (Tecan Infinite M200, Tecan Austria, Austria) after the material rested for 10 min. 

### 2.8. Histopathology of Liver Tissues

Fresh liver tissues were embedded in OCT compound (Tissue-Tek 4583, Sakura Finetek, Torrance, CA, USA), then sectioned at 4 *μ*m by use of a cryostat microtome (Leica CM3050S, Leica Microsystems, Nussloch GmbH, Nussloch, Germany), and stained with periodic acid Schiff (PAS) as we described [18]. Specimens were photographed using a SPOT Idea 3MP camera on an Olympus CKX41 inverted microscope.

### 2.9. Statistical Analysis 

Data are expressed as mean ± SEM and analyzed by one-way AVOVA with SAS version 9.0 (SAS Inst., Cary, NC). *P* < 0.05 was considered statistically significant. A Cochran-Armitage test was used for dose-effect trend analysis. 

## 3. Results

### 3.1. Identification of Chemical Constituents of *A. camphorata*

**Figure 1:**
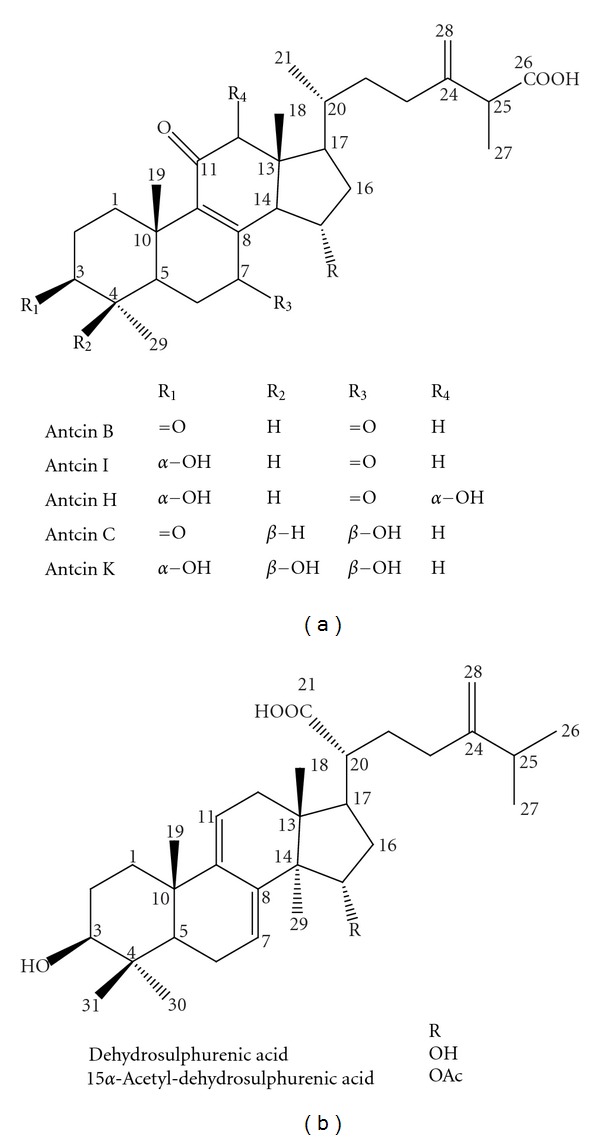
Chemical structure of identified triterpenoids from *Antrodia camphorata. *

Repeated-column chromatography of EtOH extracts of the fruiting body of *A. camphorata* on highly porous polymer gel and silica gel revealed 7 compounds. The structures were elucidated by spectroscopic analysis and compared with the literature [14].  Figure 1 shows the predominant constituents, identified as antcin B, C, H, I, K (ergostane-type triterpenoids) and dehydrosulphurenic acid, 15*α*-acetyl-dehydrosulphurenic acid (lanostane-type triterpenoids).

### 3.2. Effect of *A. camphorata* on Mouse Forelimb Grip Strength

**Figure 2:**
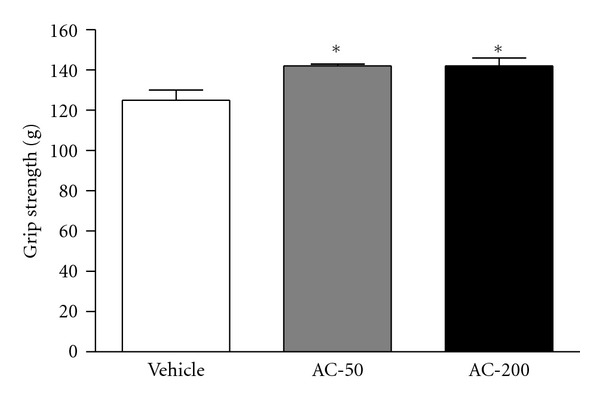
Effect of *A. camphorata* (AC) supplementation on forelimb grip strength in mice. Male ICR mice were pretreated with vehicle or 50 or 200 mg/kg ethanol extract of AC fruiting body (AC-50 or AC-200) for 7 days and then 1 h later underwent a low-force grip-strength test. Data are mean ± SEM (*n* = 10 mice). *P < 0.01 compared to vehicle control.

The grip strength of mice in the vehicle, AC-50, and AC-200 groups was 125 ± 5, 142 ± 1, and 142 ± 4 g, respectively (Figure 2), which was significantly higher, by 1.13-fold (*P* = 0.005), with AC than vehicle treatment. Grip strength was increased dose dependently with AC but not significantly (*P* = 0.066).

### 3.3. Effect of *A. camphorata* on Mouse Exercise Capacity by Forced Swim Test

**Figure 3:**
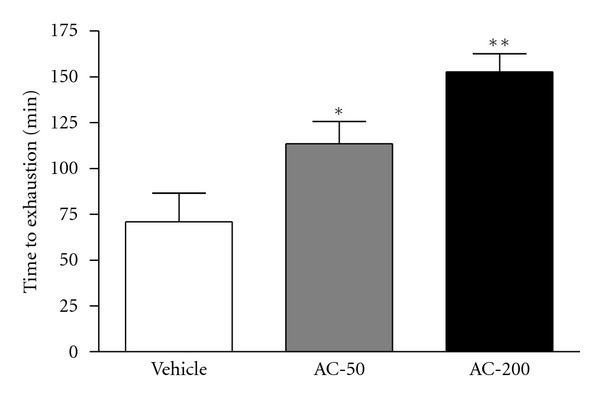
Effect of AC on swim exercise performance in mice. Mice were pretreated with vehicle, AC-50, or AC-200 for 7 days and then 1 h later underwent an exhaustive swimming test with a 5% body-weight load attached to the mouse tail. Data are mean ± SEM (*n* = 10 mice). *P < 0.05, **P < 0.001 compared to vehicle control.

The endurance of mice administered vehicle, AC-50, and AC-200 was 70.9 ± 15.7, 113.6 ± 12.1, and 152.8 ± 9.8 min, respectively (Figure 3). The swimming time was significantly longer by 1.60- (*P* = 0.016) and 2.15-fold (*P* < 0.0001) with AC-50 and AC-200, respectively, than vehicle treatment. Endurance was greater with AC-200 than AC-50 treatment (*P* < 0.05). Trend analysis revealed a significant dose-dependent effect with vehicle, AC-50, and AC-200 on swimming time (*P* < 0.0001). 

### 3.4. Effect of *A. camphorata* on Mouse Plasma Lactate, Ammonia, and Glucose Levels and CK Activity with Exercise 

**Figure 4:**
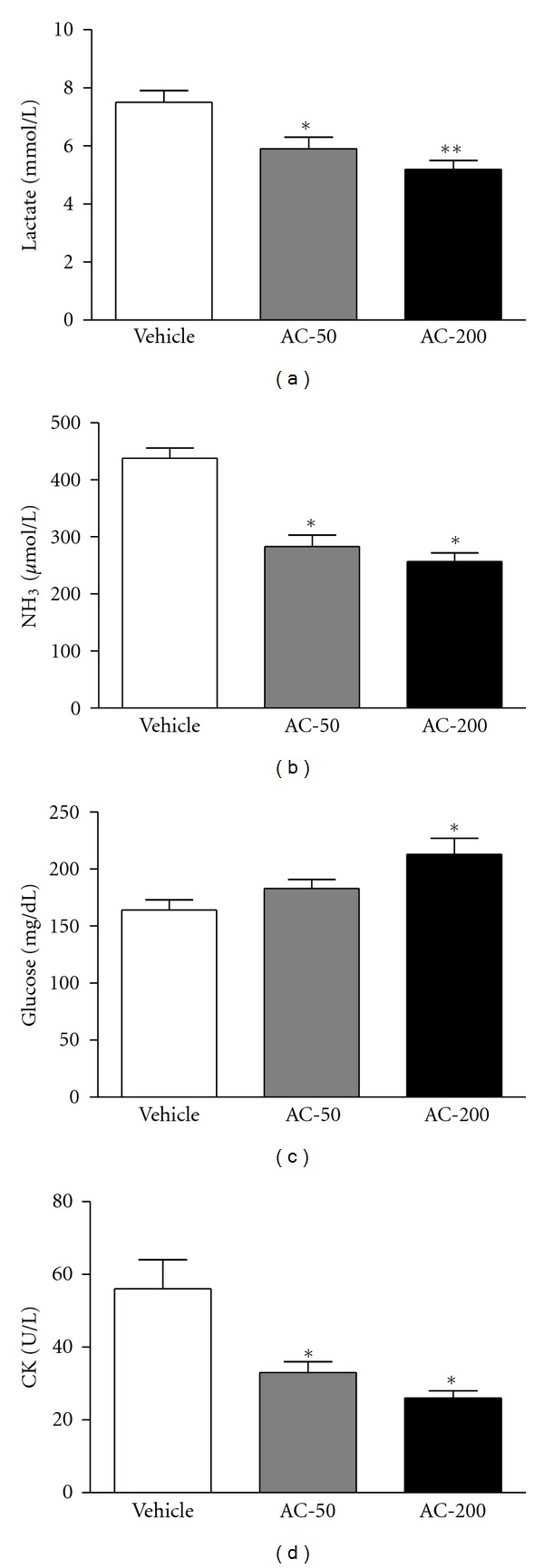
Effect of AC on plasma lactate, ammonia, and glucose levels and creatine kinase (CK) activity after exercise. Mice were pretreated with vehicle, AC-50, or AC-200 for 7 days and then 1 h later underwent a 15 min swim test. Data are mean ± SEM (*n* = 10 mice). (a) Lactate: *P < 0.005, **P < 0.0001 compared to vehicle control. (b) Ammonia: *P < 0.0001 compared to vehicle control. (c) Glucose: *P < 0.05 compared to vehicle control. (d) CK: *P < 0.005 compared to vehicle control.

Lactate levels in the vehicle, AC-50 and AC-200 groups were 7.5 ± 0.4, 5.9 ± 0.4, and 5.2 ± 0.3 mmol/L, respectively (Figure 4(a)), and were significantly lower, by 21% (*P* = 0.0046) and 31% (*P* < 0.0001), with AC-50, and AC-200, respectively, than vehicle treatment. 

Plasma ammonia levels in the vehicle, AC-50, and AC-200 groups were 438 ± 18, 283 ± 20, and 257 ± 15 *μ*mol/L, respectively (Figure 4(b)), and were significantly lower, by 35% and 41%, with AC-50 and AC-200, respectively, than vehicle treatment (*P* < 0.0001). 

Plasma glucose content in the vehicle, AC-50, and AC-200 groups was 164 ± 9, 183 ± 8, and 213 ± 14 mg/dL, respectively (Figure 4(c)), and was significantly higher, by 1.3-fold (*P* = 0.0035), with AC-200 than vehicle treatment. 

Plasma CK activity, a muscular damage marker, in the vehicle, AC-50, and AC-200 groups was 56 ± 8, 133 ± 3 and 26 ± 2 U/L, respectively (Figure 4(d)), and was significantly lower, by 41% (*P* = 0.0038) and 54% (*P* = 0.0003), with AC-50 and AC-200, respectively, than vehicle treatment. Trend analysis revealed that AC treatment had a significant dose-dependent effect on increasing blood glucose (*P* = 0.0003) content and decreasing plasma lactate and ammonia levels and CK activity (all *P* < 0.0001).

### 3.5. Effect of *A. camphorata* on Mouse Muscular and Hepatic Glycogen Levels

**Figure 5:**
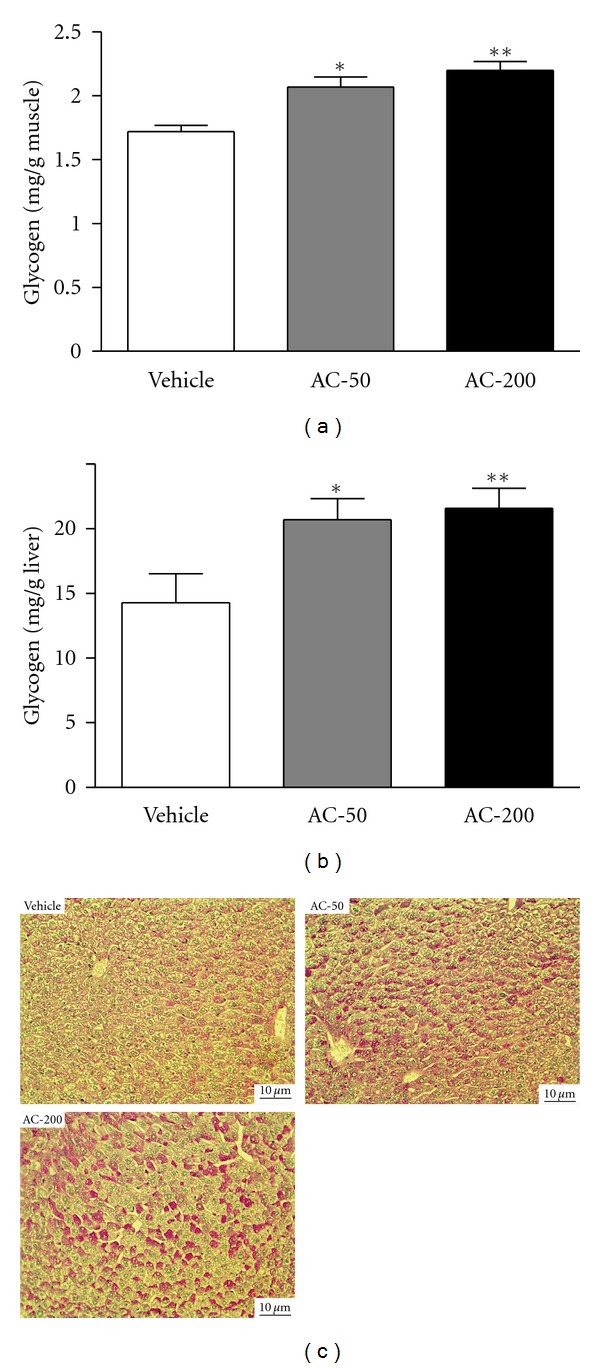
Effect of AC on muscular and hepatic glycogen levels. Mice were pretreated with vehicle, AC-50, or AC-200 for 7 days and then killed 1 h later. Glycogen levels in muscle and liver tissues were determined. Data are mean ± SEM (*n* = 10 mice). (a) Skeletal muscle glycogen: *P < 0.005, **P < 0.0001 compared to vehicle control. (b) Liver glycogen: *P < 0.05, **P < 0.005 compared to vehicle control. (c) Light micrographs of liver tissue hepatocytes stained with periodic acid-Schiff (PAS) (magnification ×200; scale bar, 10 *μ*m).

Muscular glycogen levels in the vehicle, AC-50, and AC-200 groups were 1.72 ± 0.05, 2.07 ± 0.08, and 2.20 ± 0.21 mg/g skeletal muscle, respectively (Figure 5(a)), and were significantly higher, by 1.20- (*P* = 0.0010) and 1.28-fold (*P* < 0.0001), with AC-50 and AC-200, respectively, than vehicle treatment. 

Hepatic glycogen levels in the vehicle, AC-50, and AC-200 groups were 14.28 ± 2.26, 20.70 ± 1.64, and 20.70 ± 1.64 mg/g liver, respectively (Figure 5(b)), and were significantly higher, by 1.45- (*P* = 0.0213) and 1.51-fold (*P* = 0.0098), with AC-50 and AC-200, respectively, than vehicle treatment. Trend analysis revealed that AC treatment had a significant dose-dependent effect on increasing muscular (*P* < 0.0001) and hepatic glycogen levels (*P* = 0.0034). 

PAS staining is used to detect glycogen in tissues. Liver sections with AC-50 and AC-200 treatment showed a significantly greater number of PAS-positive hepatocytes than those with vehicle treatment, which suggests glycogen accumulation in AC-50 and AC-200 hepatocytes (Figure 5(c)). Thus, AC treatment significantly increased glycogen deposition in liver tissues.

## 4. Discussion

Many natural sources have been studied as supplements to improve fatigue symptoms. These studies focused on plant extracts and emphasized the importance of phytocompounds such as polysaccharides [19], flavonoids [20], and peptides [7]. In this study, we compared the fatigue-alleviating effects of 2 doses of *A. camphorata* and vehicle on endurance in exercised and weight-loading mice. Ethanol extract of *A. camphorata* fruiting bodies dose-dependently enhanced exercise performance and reduced muscle fatigue physiological indexes; so *A. camphorata* may have ergogenic and antifatigue functions.

The energy metabolism of muscular activity determines the level of physiological fatigue [21]. Exercise endurance is an important variable in evaluating antifatigue treatment. In our mice, forelimb grip strength was significantly elevated, by 1.13-fold with both AC doses. Regulatory training programs are needed for grip strength elevation, so we observed no difference between the doses. However, in the exhaustive physical-exercise swim test, maximal swimming time was increased dose-dependently with the AC doses (Figure 2). Both results show an elevation in exercise endurance in mice, which suggests that *A. camphorate* ethanol extract has antifatigue effects.

Biochemical variables, including lactate, ammonia (NH_3_), glucose, and CK, are important indicators of muscle fatigue after exercise [22]. The muscle produces a great quantity of lactate when it obtains enough energy from anaerobic glycolysis during high-intensity exercise. The increased lactate level further reduces pH value, which could induce various biochemical and physiological side effects, including glycolysis and phosphofructokinase and calcium ion release, through muscular contraction [23]. Ammonia, the metabolite of protein and amino acid, was linked to fatigue as early as 1922 [24]. The increase in ammonia in response to exercise can be managed by the use of amino acids or carbohydrates that interfere with ammonia metabolism [25]. The increase in ammonia level is related to both peripheral and central fatigue during exercise. Energy storage and supply is another important factor related to exercise performance. In terms of energy expenditure with exercise, rapid ATP consumption and energy deficiency is a critical cause of physical fatigue [26]. Skeletal muscle mainly catabolizes fat and carbohydrates as sources of energy during exercise. Glycogen is the predominant source of glycolysis for ATP production. Therefore, glycogen storage directly affects exercise ability [27]. After we administered AC or vehicle to mice for 7 days, serum lactate and ammonia levels were notably lower with AC than vehicle treatment after the swim test. Therefore, AC should enhance lactate and ammonia elimination. Additionally, both hepatic and muscular glycogen levels were increased in response to AC treatments. These results illuminate the release of glucose from tissue glycogen for energy recovery with AC and the statistical significance on trend analysis after exercise.

CK, a muscular damage index, was significantly ameliorated dose-dependently with AC treatment on trend analysis. High-intensity exercise challenge could physically or chemically cause tissue damage. It can cause sacromeric damage and muscular cell necrosis [28]. The cells release specific proteins such as CK and myoglobin into the blood as muscular damage indexes. Clinically, CK is assayed in blood tests as a marker of myocardial infarction, rhabdomyolysis (severe muscle breakdown), muscular dystrophy, autoimmune myositides, and acute renal failure [28].

To clarify the components contributing to the AC antifatigue function, we separated AC extracts to identify the index compounds and found a great proportion of triterpenoid compounds. We identified about 78 compounds, including polysaccharides, benzenoids, diterpenes, triterpenoids, steroids, and maleic/succinic acid derivatives [9]. Previous studies demonstrated triterpenoids as the predominant constituents in fruiting bodies of *A. camphorata*, with its bioactivity widely studied in antitumor research [10]. Triterpenoids are an important bioactive class of natural products with diverse structures. They are synthesized from isopentenyl pyrophosphate through the 30-carbon intermediate squalene; the major skeleton types include oleananes, ursanes, lupanes, protostanes, lanostanes, and dammaranes [29]. More than 20,000 compounds have been reported. Although their functions and promising biological properties have received considerable attention, studies of the relationship of triterpenoids and antifatigue activity are still limited. Pentacyclic triterpenoid-enriched extracts from Chinese bamboo shavings were recently shown to have antifatigue activities [30]. We found ergostane and lanostane skeleton triterpenoids in the bioactive *A. camphorata* extract. Ergostane-type triterpenoids are specific to the fruiting body of *A. camphorata*. These ergostane and lanostane triterpenoids may therefore be important antifatigue active components in *A. camphorata*. 

In conclusion, ethanol extracts of the fruiting body of *A. camphorata* have antifatigue activity by decreasing plasma lactate and ammonia levels and increasing blood glucose concentration and liver and muscle glycogen deposition, thereby elevating exercise performance in mice. We identified putative bioactive components in these extracts. Although the detailed antifatigue mechanisms of *A. camphorata* remain to be elucidated, this study provides science-based evidence to support traditional claims of antifatigue results with *A. camphorata* treatment and suggests a use for *A. camphorata* as an ergogenic and antifatigue agent.
